# Supplementary material for: Multiomic Integration Reveals Taxonomic Shifts Correlate to Serum Cytokines in an Antibiotics Model of Gut Microbiome Disruption
Source: Cell Mol Bioeng. 2025 Aug 24;18(5):369–85. doi: 10.1007/s12195-025-00861-2 (PMC12579621; doi:10.1007/s12195-025-00861-2)
Supplement: Supplementary file 1 — Supplementary file1 (PDF 178 kb) [file 12195_2025_861_MOESM1_ESM.pdf]

Table S1: Multiomic integration effectively classified antibiotic treatments. *Area under the curve (AUC) for Data Integration Analysis for Biomarker discovery using Latent cOmponents (DIABLO) and associated p-value determined using mixOmics R package.*

Latent Variable	Block	Comparison	AUC	P value
LV 1	Genera	Amp vs. others	0.80	0.004
		Neo vs. others	0.81	0.004
		Amp+Neo vs. others	0.98	<0.001
	Cytokines	Amp vs. others	0.66	0.14
		Neo vs. others	0.74	0.03
		Amp+Neo vs. others	0.81	0.001
LV 2	Genera	Amp vs. others	0.95	<0.001
		Neo vs. others	0.89	<0.001
		Amp+Neo vs. others	0.99	<0.001
	Cytokines	Amp vs. others	0.90	<0.001
		Neo vs. others	0.81	0.003
		Amp+Neo vs. others	0.80	0.001

Table S2: Serum cytokine concentrations are different with respect to control. *Serum cytokine concentrations reported as mean ± SEM. Bold text denotes a statistically significant difference (p < 0.05) compared to control. Tested with Kruskal Wallis and subsequent Dunn testing with Benjamini-Hochberg correction.*

Cytokine	Control	Amp	Amp+Neo	Neo
Eotaxin	2182.3 ± 264.9	2656.9 ± 35	2039.3 ± 157.8	**3104.4 ± 373.9**
Granulocyte-colony stimulating factor (G-CSF)	572.3 ± 402.9	708.1 ± 344.8	422.4 ± 137.7	624.2 ± 462.8
Interferon gamma (IFNγ)	12.4 ± 8.5	10.0 ± 6.8	16.4 ± 13.5	8.4 ± 3.6
Interleukin (IL)-10	45.4 ± 25.3	17.2 ± 14.2	43.0 ± 14.4	48.8 ± 25.7
IL-12(p40)	70.3 ± 56.5	38.1 ± 39.1	73.1 ± 40.6	**56.6 ± 55.9**
IL-13	136 ± 59.5	109.5 ± 40	158.1 ± 47.3	142.9 ± 46.1
IL-1β	7.2 ± 4.6	7.4 ± 5	8.0 ± 3.9	7.4 ± 4.3
IL-1a	356.3 ± 337.3	618.2 ± 249.1	437.1 ± 286.4	**822.7 ± 768.9**
IL-2	10.6 ± 6.6	7.5 ± 4.7	7.6 ± 3.4	8.1 ± 4.1
IL-3	5.4 ± 4.8	7.8 ± 7.5	3.0 ± 2.9	4.9 ± 4.2
IL-5	33.6 ± 17.9	39.7 ± 38.8	27.9 ± 17.7	26.9 ± 14.0
IL-6	12.7 ± 7.2	6.6 ± 7.2	**23.1 ± 13.0**	21.5 ± 21.3
IL-7	6.7 ± 4.4	5.7 ± 2.7	4.5 ± 2.6	7.3 ± 4.3
IL-9	217.7 ± 112.9	294.2 ± 202.6	227.5 ± 109.0	202.5 ± 135.0
Interferon-gamma induced protein (IP-10)	411.0 ± 266.4	559.2 ± 125.8	418.3 ± 170.4	**602.5 ± 254.8**
Keratinocyte chemoattractant (KC)	117.8 ± 72.0	161.4 ± 54.6	131.8 ± 43.5	**186.2 ± 60**
Leukemia Inhibitory Factor (LIF)	11.0 ± 8.7	8.6 ± 4.6	**19.7 ± 20.7**	9.8 ± 5.3
Lipopolysaccharide-induced CXC chemokine (LIX)	3161.1 ± 1894.2	**4514.6 ± 528.4**	2705.9 ± 1451.4	**4246.5 ± 1935.1**
Macrophage colony-stimulating factor (M-CSF)	42.0 ± 43.7	13.4 ± 12.8	27.9 ± 21.3	30.5 ± 21.8
Macrophage chemoattractant protein (MCP-1)	85.9 ± 86.8	64.2 ± 36.4	37.4 ± 34.4	48.4 ± 33.2
Monokine induced by gamma (MIG)	1274.6 ± 861.7	1311.4 ± 517.2	1244 ± 669.1	1365.4 ± 87.9
Macrophage inflammatory protein (MIP-1β)	64.3 ± 45.6	**8.2 ± 4.8**	43.6 ± 15.3	49.7 ± 16.6
MIP-2	481.7 ± 309.3	629.6 ± 203.1	374.7 ± 188	**620.4 ± 283.0**
Regulated on Activation, Normal T Expressed and Secreted (RANTES)	56.9 ± 49.7	**70.5 ± 16.9**	45.2 ± 25.6	71.3 ± 30.0
Tumor Necrosis Factor alpha (TNFα)	13.2 ± 11.6	15.2 ± 9.5	7.8 ± 5.9	8.2 ± 4.2
Vascular endothelial growth factor (VEGF)	6.9 ± 5.9	9.9 ± 2.1	4.2 ± 4.4	**8.3 ± 4.8**

Table S3: Cytokines with significant correlation to taxa relative abundance are also associated with gut and joint physiologic and pathophysiologic processes.

Cytokine	Gut Inflammation	Bone Restitution	Cartilage Degradation
IL-13	Donlan, *et al.*, 2024 [90]	Onoe, *et al.*, 1996 [91]	Nabbe, *et al.*, 2005 [92]
IL-6	Shahini, *et al.*, 2023 [93]	Yoshitake, *et al.*, 2008 [32]<br>Ishimi, *et al.*, 1990 [31]	Flannery, *et al.*, 2000 [94]
IL-10	Kuhn, *et al.*, 1993 [95]	Dresner-Pollak, *et al.*, 2004 [96]	Behrendt, *et al.*, 2018 [97]<br>Kasama, *et al.*, 1995 [98]
MIP-2	Ohtsuka, *et al.*, 2001 [99]	Ha, *et al.*, 2011 [100]	Kasama, *et al.*, 1995 [98]
MIP-1B	Lillard, *et al.*, 2003 [89]<br>Grimm, *et al.*, 1996 [101]	Abe, *et al.*, 2002 [34]	Stucker, *et al.*, 2025 [36]
LIX	Kwon, *et al.*, 2005 [102]	Klosterhoff, *et al.*, 2022 [103]<br>Sundaram 2013 [104]	Kawata, *et al.*, 2021 [105]
VEGF	Scaldaferri, *et al.*, 2009 [106]	Hu, *et al.*, 2016 [107]	Nagao, *et al.*, 2017 [108]
Eotaxin	Coburn, *et al.*, 2013 [109]	Ahmadi, *et al.*, 2020 [110]	Hsu, *et al.*, 2004 [111]
RANTES	Grimm, *et al.*, 1996 [101]	Lechner, *et al.*, 2018 [112]	Alaaeddine, *et al.*, 2001 [113]
